# Adequacy of Anesthesia Guidance for Colonoscopy Procedures

**DOI:** 10.3390/ph14050464

**Published:** 2021-05-14

**Authors:** Michał Jan Stasiowski, Małgorzata Starzewska, Ewa Niewiadomska, Seweryn Król, Kaja Marczak, Jakub Żak, Aleksandra Pluta, Jerzy Eszyk, Beniamin Oskar Grabarek, Izabela Szumera, Michał Nycz, Anna Missir, Lech Krawczyk, Przemysław Jałowiecki

**Affiliations:** 1 Department of Emergency Medicine, Faculty of Medical Sciences in Zabrze, Medical University of Silesia, 40-555 Katowice, Poland; sernik7@gmail.com (J.Ż.); apluta@autograf.pl (A.P.); iza_sz@vp.pl (I.S.); lech.kraw@gmail.com (L.K.); olaf@pro.onet.pl (P.J.); 2 Department of Anaesthesiology and Intensive Therapy, 5th Regional Hospital, Medykow Square 1, 41-200 Sosnowiec, Poland; seweryn.krol@gmail.com (S.K.); kaja.marczak.wss5@gmail.com (K.M.); aniami521@interia.pl (A.M.); 3 Department of Cardiac Anaesthesia and Intensive Care, Silesian Centre for Heart Diseases, 41-800 Zabrze, Poland; starzewskam@gmail.com; 4 Department of Epidemiology and Biostatistics, School Health Sciences in Bytom, Medical University of Silesia, Piekarska 18 Street, 41-902 Bytom, Poland; e.j.niewiadomska@gmail.com; 5 Department of General, Colorectal and Polytrauma Surgery, Faculty of Health Sciences in Katowice, Medical University of Silesia, 40-555 Katowice, Poland; michalt.nycz@gmail.com; 6 Unit of Endoscopy by the Department of Gastroenterology, 5th Regional Hospital, Medykow Square 1, 41-200 Sosnowiec, Poland; jeszyk@wp.pl; 7 Department of Histology, Cytophysiology and Embryology, Faculty of Medicine in Zabrze, University of Technology in Katowice, 41-800 Zabrze, Poland; bgrabarek7@gmail.com; 8 Department of Nursing and Maternity, High School of Strategic Planning in Dąbrowa Górnicza, 41-300 Dąbrowa Górnicza, Poland

**Keywords:** adequacy of anesthesia (AoA), state entropy (SE), response entropy (RE), surgical pleth index (SPI), colonoscopy procedure (CP), postoperative nausea and vomiting (PONV), intraprocedural awareness with recall (IAwR), postprocedural pain (PPP), propofol, fentanyl (FNT), intravenous sedoanalgesia (ISA)

## Abstract

In patients undergoing colonoscopy procedures (CPs), inadequate dosing of hypnotic drugs (HD) and opioid analgesics (OA) during intravenous sedoanalgesia (ISA) may lead to intraprocedural awareness with recall (IAwR), intraprocedural (IPP) and postprocedural pain (PPP), as well as postoperative nausea and vomiting (PONV). The aim of this study was to evaluate whether the titration of HD and OA based on the observance of changing values of state entropy (SE) and surgical pleth index (SPI) (adequacy of anesthesia—AoA), state entropy alone, or standard practice may reduce the number of adverse events. One hundred and fifty-eight patients were included in the final analysis. The rate of IAwR and IPP was statistically more frequent in patients from the C group in comparison with the AoA and SE groups (*p* < 0.01 and *p* < 0.05, respectively). In turn, the rate of PPP, PONV, and patients’ and operators’ satisfaction with ISA between groups was not statistically significant (*p* > 0.05). Changes in hemodynamic parameters, demand for HD, and OA were statistically significant, but of no clinical value. In patients undergoing CPs under ISA using propofol and FNT, as compared to standard practice, intraprocedural SE monitoring reduced the rate of IAwR and IPP, with no influence on the rate of PPP, PONV, or patients’ and endoscopists’ satisfaction. AoA guidance on propofol and FNT titration, as compared to SE monitoring only, did not reduce the occurrence of the aforementioned studied parameters, imposing an unnecessary extra cost.

## 1. Introduction

Endoscopic procedures of the gastrointestinal tract are associated with considerable patient discomfort and pain [1,2,3]. American guidelines for intravenous sedoanalgesia (ISA) in gastrointestinal endoscopy highlight the need to provide safe, painless, and anxiety-free procedures for patients, which involves sedation whenever needed [2]. For that reason, the number of sedated colonoscopy procedures (CPs) is increasing [3]. However, it is worth mentioning that the need for ISA during CPs in the literature is still a matter of debate [4]. While ISA for CPs is a standard procedure in some countries, in others an unsedated procedure is preferred. While controversies remain, the fact is that, in some groups of patients, sedation will always be the method of choice [5], which is especially true for patients who are younger, female, and characterized as having a difficult CP, specific indications, cardiopulmonary complications, or using opioids or benzodiazepines [6].

One of the most common reasons for patients’ low satisfaction scores during the postprocedural period is an unacceptable perception of acute postprocedural pain (PPP) [7] and postoperative nausea and vomiting (PONV) [8], which may be exacerbated by inadequate opioid dosing [9]. 

Therefore, numerous anesthetic modalities are employed to reduce PPP intensity and PONV, which result in patients’ and endoscopists’ low satisfaction scores [10]. Intraprocedural titration of propofol during CPs is a widely accepted anesthetic technique providing satisfactory sedation [11], superior to midazolam alone [12]. Numerous anesthesia techniques, such as intraprocedural opioid titration [13], intravenous lidocaine infusion [14], an addition of a low dose of intravenous ketamine [15], and inhalation of an anesthetic mixture of oxygen with nitrous oxide [16], are employed to alleviate the visceral pain associated with the performance of CPs. In order to optimize the depth of anesthesia, monitoring has been introduced into anesthesiology practices. The most widely used are the Bispectral Index (BIS) and a State and Response Entropy EEG (SE & RE EEG) [17,18,19,20]. In recent years, the Surgical Pleth Index (SPI), a new method of objective assessment of nociception/antinociception balance, has been added to RE and SE order to serve together as the Adequacy of Anesthesia (AoA) concept, a new tool (SPI; GE Healthcare, Helsinki, Finland) that, with different success rates, has been proven useful to guide adequate anesthetic dosages for hypnosis as well as opioids for analgesia [21,22,23], aiming to reduce the occurrence of unwelcome adverse events [24,25,26].

Considering all of the above, this study was designed to evaluate whether overall propofol/fentanyl (FNT)-based ISA using conventional techniques for their titration as compared to SE monitoring for sedation using propofol with conventional techniques for FNT titration or AoA monitoring to guide propofol and FNT ISA can reduce the occurrence of adverse events in patients undergoing CPs. Additionally, we analysed which of the aforementioned anesthetic techniques may optimize the use of resources and result in patients’ and endoscopists’ higher satisfaction scores regarding patients undergoing CPs—in the current literature, this has not yet been studied.

## 2. Results

Out of 166 patients assessed for eligibility, 158 were included in the final analysis (Figure S1 and Table S1; *p* > 0.05). 

Before the induction of ISA, differences in the values of the monitored parameters between the studied groups were not statistically significant (*p* > 0.05). During the second stage of the study, during the CP, statistically significant differences between the studied groups were found regarding mean values of arterial blood pressure. Mean values of SAP were statistically significantly lower in patients allocated to the SE and AoA groups, compared to the control group. There were no statistically significant differences in any of the mean values of monitored parameters between the AoA and SE groups. 

During observation in the postanesthesia care unit (PACU), after the CP, statistically significant differences between the studied groups regarding mean values of arterial blood pressure were found. The mean values of SAP and MAP were statistically significantly lower in patients allocated to the AoA group, compared to the control group. No statistically significant differences between the mean values of studied parameters in the PACU in patients allocated to the AoA and SE groups were found (see Table 1). 

In order to optimize resources, the consumption of FNT and propofol in the studied groups of patients and the length of the CPs were studied. 

Consumption of FNT proved to be statistically significantly higher in patients allocated to the AoA group than in Group C and Group SE. On the other hand, consumption of propofol proved to be statistically significantly higher in Groups AoA and SE, compared to the control group. Intraprocedural demand for rescue crystalloid infusion, necessity of intraprocedural rescue atropine, and ephedrine administration did not prove to be statistically different between groups (Table 2). 

Perioperative variations of values of monitored hemodynamic parameters (maximum, minimum, and mean values of HR, SAP, MAP, and DAP) constituting patients’ perioperative safety were also studied, and statistically significant differences between groups were observed (Table 3). During the CP (Stage 2), maximum values of SAP were statistically significantly lower in both studied groups compared to the control group. Maximum values of MAP were statistically significantly lower in the AoA group compared to the control group. Mean values of SAP, MAP, and DAP were statistically significantly lower in the AoA group compared to the control group. Postoperatively, during observance in the PACU, statistically significantly lower values of minimum SAP and MAP in patients allocated to the AoA group in comparison to the control group were found. Interestingly, no statistically significantly differences were observed regarding the values of monitored parameters between Groups SE and AoA (see Table 4).

No statistically significant differences were observed in terms of APFEL scores, the rate of PONV, or the rate of postoperative perception of acceptable (NRS < 4) and unacceptable (NRS > 3) PPP between the studied groups (Table 3).

**Figure 1:**
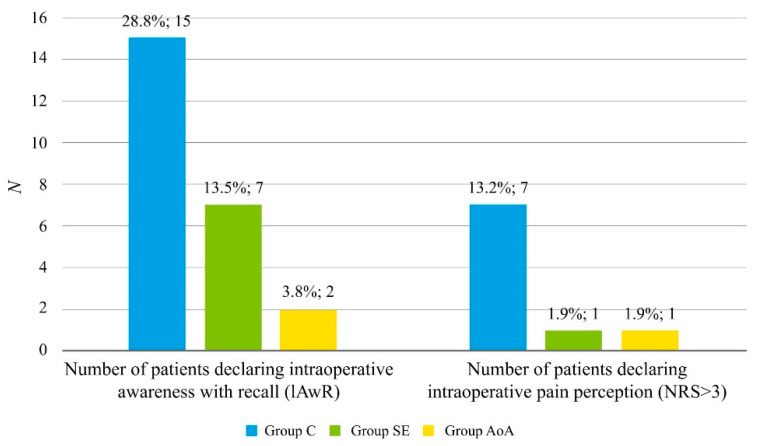
Rate of adverse events in patients allocated to the studied groups, specifically the incidence of intraoperative awareness with recall (IAwR) and intraoperative pain perception. Results are presented as numbers (percentages) for nominal variables. *p*-values were found by an X^2^ test for nominal variables. A: Significantly more in Group C than in Groups SE and AoA (*p* < 0.05 for both); Abbreviations: Group C: control group; Group SE: Entropy SE group; Group AoA: Adequacy of Anesthesia group; NRS: Numeric Pain Rating Scale; IAwR: intraoperative awareness with recall.

Both patients and endoscopists were surveyed to analyze their satisfaction with the performed ISA. No statistically significant differences regarding their level of satisfaction with the quality of the performed ISA, despite the group allocation, were found (see Table 5). Nevertheless, the number of patients declaring unacceptable IPP (NRS > 3) using a numeric rating scale (NRS) was higher among patients allocated to the control group compared to the two other groups, which was statistically significant. Additionally, the number of patients declaring IAwR was higher among patients allocated to the control group, compared to the two other groups, which was statistically significant. No statistically significant differences regarding the rate of IAwR and unacceptable IPP were found between the AoA and SE groups. Both patients’ and endoscopists’ satisfaction with the performed anesthesia was similar in all studied groups (Figure 1).

Traditionally, inadequate intraprocedural analgesia was detected by observation of a sudden intraprocedural increase in HR and MAP, whereas a recent vasoactive reaction to intraprocedural surgical stimulation was detected by observing changes in SPI values. Correlations between traditional methods of assessing inappropriate intraprocedural vasoactive reactions (increases in HR and MAP) on a surgical stimulation with increases in SPI values were also analyzed. No statistically significant, positive correlation was found between maximum SPI values and maximum values of HR and MAP or between minimum SPI values and minimum MAP and HR values in the second stage of the study (Table 5).

## 3. Discussion

The utility of ISA for CPs is a widely recognized anesthetic regimen based on titration of both HD and OA to ensure proper reversible hypnosis and alleviation of pain, despite the possibility of respiratory depression, dose-dependent hypotension, and pain during injection—which are rare but possible coexisting adverse events. Thanks to its rapid onset (<1 min) and short duration (approximately 4–8 min), propofol is the most popular intravenous HD, used alone during CPs or in combination with other drugs like fentanyl, which is the most popular OA with rapid onset (about 2 min) and a short duration (approximately 30 min) [27]. 

Monitoring the depth of sedation and general anesthesia has become increasingly popular, despite its drawbacks [17,28,29]. Principally, changes in electroencephalographic patterns, which are caused by the administration of intravenous anesthetics, like propofol in the current study, or widely used volatile anesthetics during general anesthesia, evaluate cerebral patients’ electrical activity [30,31]. Therefore, depth-of-anesthesia monitors, which are based on the processed electroencephalographic signal, transform it into a simple digital score that corresponds to the patient’s level of consciousness during general anesthesia in order to control the depth of the hypnotic component [32]. 

SE score, which reflects the patient’s cortical state during different stages of general anesthesia, is computed from the electroencephalographic signal only, whereas the response entropy (RE) index is computed from both electroencephalography and electromyography. The EEG signal is transformed into a digital SE score between 0 and 91, which is visible on the anesthetic monitor. Thus, it is easy to control the hypnotic component of general anesthesia by, for example, the titration of HD, because a value of 91 indicates an awake state; between 60 and 80 indicates deep to mild sedation; between 40 and 60 indicates a level of unconsciousness suitable for surgery; between 30 and 40 indicates too deep an anesthesia; and under 30 indicates an overdose of anesthetics. Moreover, the placement of a frontal electrode is easy and does not entail complex, time-consuming preoperational preparations [16,17]. 

There are few studies regarding the monitoring of the depth of intravenous analgesia for gastrointestinal endoscopy, but the results are contradictory. 

Different methods of instrumental evaluation of the efficacy of nociception/antinociception balance and OA titration to alleviate the IPP during anesthesia are gaining popularity [24,33]. Soral et al. [33] compared the effects of analgesia management with ANI-guided analgesia versus analgesia guided with conventional methods induced with propofol and ketamine and continued using infusion of propofol and remifentanil in patients undergoing a CP. They observed reduced OA consumption when ANI monitoring was used and concluded that patient safety was improved. This observation was dissimilar to the finding from the current study showing no influence of an addition of SPI on SE for guidance of propofol/FNT ISA, but they found an advantage over conventional methods based on anesthesiologists’ intuition and the observance of hemodynamic changes. Contrary to the current study, they monitored the depth of analgesia using BIS scores in both groups; although numerous studies do not show its usefulness in sedation guidance in CPs, the BIS scores between groups were equal, having no impact on the intraprocedural vasoactive reaction to operators’ manipulations. In the current study, SE-based guidance to titrate propofol was used, and the values between SE and AoA groups also did not differ statistically, so the quality of the titration of propofol between these two groups was also comparable. Nevertheless, the utility of SPI guidance for FNT administration proved to be of no benefit in the AoA group compared to the SE group because the rate of adverse events did not differ statistically. The difference between the current study and the findings of Soral et al. may be due to the different OA used because the precision of remifentanil administration due to its ultrashort onset of action (<2 min), compared to FNT with its short onset of action (<5 min), may be, to a greater extent, responsible for the final outcomes [33]. 

To the best of our knowledge, in the current literature, there is only one report regarding the utility of SPI guidance for OA administration in patients undergoing sedation for upper endoscopic procedures to alleviate visceral pain, as compared to conventional methods [33]. Statistically significant differences were found regarding the rate of hiccupping and cough, as well as SPI values, in patients receiving propofol only, compared to propofol/remifentanil under SPI guidance, with no influence on hemodynamic stability and endoscopists’ satisfaction. The authors concluded that SPI guidance on the speed of infusion of propofol/remifentanil indicates an advantage over the infusion of propofol alone for upper endoscopic procedures. Nevertheless, the authors underlined that only 30 patients were allocated to each group, which could have markedly influenced the outcomes, in comparison to over 50 in the current study.

SPI is derived from the photoplethysmographic waveform amplitude and the beat-to-beat interval of the heart [34]. Changes in SPI value were reported to correspond to serum OA concentration [19], so SPI guidance seems to be an excellent tool for the administration of intraprocedural rescue OA because fluctuations on the monitor in a digital form (0: no pain; 100: maximum pain) ease its intraprocedural use and interpretation [35]. In the current study, no statistically significant, positive correlation of increase in SPI and HR values was found. Moreover, no correlation of increase in MAP and SPI values was observed, which was statistically significant, possibly due to the fact that SPI fluctuates online and is recorded every minute, similar to HR, whereas MAP is measured every 5 min. We suppose that, taking into consideration the relatively short length of the procedure, MAP, SAP, and DAP value registration every 5 min was potentially too rare because the titration of rescue OA with a short time of onset of action, below 5 min, might have influenced the final outcomes, as a potential increase in value in hemodynamic parameters was overlooked in some cases. Similar findings to those of the current study led Gruenewald et al. [19] to conclude that a vasoactive reaction to nociceptive stimulation resulted in [36] in an increase in SPI value, which was also dependent on OA serum concentration, whereas such a simultaneous effect was hardly reflected in the values of HR, SE, and RE increase after nociceptive stimulation. Therefore, further studies are required to investigate the utility of observing SPI values in reactions to nociceptive intraprocedural stimulation. It will be helpful to optimize both perioperative outcomes and reduce the occurrence of unwelcome adverse events [36]. 

Inappropriate intraprocedural titration of OA may lead to adverse events, such as PPP, PONV, and hemodynamic instability. Up to 80% of patients may experience moderate to high-intensity PPP [7], and 25% of patients may also experience PONV in the postprocedural period [37]. In the current study, the risk of PONV was equal in each group, as expressed by the Apfel score (Table 3), and despite the group allocation, only 3 patients out of 158 included in the final analysis suffered from PONV: two from the control group (3.8%), one from the AoA group (1.9%), and none from the SE group, which did not prove statistically significant (*p* = 0.25). Seleem et al. [38], in a study concerning different intravenous anesthesia modalities in patients undergoing CP, found that 28 out of 75 patients receiving propofol with FNT complained of PONV, as compared to 8 out of 75 patients receiving propofol with ketamine, which was statistically significant (*p* = 0.0001). In comparison to the current study findings, they achieved poorer outcomes, probably due to the fact that they titrated FNT according to the observance of hemodynamic parameters, which may not necessarily properly reflect the demand for OA [38]. Bergmann et al. [39] found no difference between the groups with regard to the rate of IawR using the anesthetic remifentanil/propofol-based modalities with SE only versus AoA guidance in outpatient arthroscopic orthopedic anesthesia. They noted no incidence of IawR, whereas in the current study, in the SE and AoA groups, the number of patients reporting IawR was 9 altogether, probably due to the target SE of around 70 in the current study and around 60 in the study of Bergmann et al. [39].

In the current study, the rate of IPP appeared to be statistically significantly higher between the control group and the two other studied groups. Interestingly, between the two studied groups—the AoA and SE groups—such a difference was not found, so the conclusion is that SPI guidance for intraprocedural OA administration does not have clinical relevance. 

Our study findings are somehow similar to those of Won et al. [40], who analyzed six randomized controlled trials comparing 463 patients. They concluded that no intergroup difference was observed in the degree of PPP or the incidence of perioperative adverse events [40]. 

Mainly, respiratory and hemodynamic instability threatens patients’ safety during sedation for CP [41]. In the current study, numerous statistically significant differences were observed between groups in terms of hemodynamic parameters at different stages of the study, but they were of no clinical relevance because they did not meet the criteria for hypotension, resulting in organ hypoperfusion in the case of excessive hypnosis and analgesia or hypertensive crisis as a result of extensive intraprocedural vasoactive reaction to operators’ nociceptive stimulation in the case of insufficient hypnosis and analgesia. Despite the group allocation, there was a necessity of intraprocedural administration of atropine due to bradycardia in only 12 patients and ephedrine due to hypotension in 2 patients, which was not statistically significant between groups. Von Delius et al. [42], in their abovementioned study monitoring the depth of low-dose midazolam and propofol sedation during CPs with either the Bispectral Index (BIS) or the A-line auditory evoked potential index (AAI), observed that, although BIS and AAI correlated with the level of sedation, hemodynamic variables were poor indicators of the hypnotic–anesthetic status of the patient. In the study of Bilgi et al. [43], under BIS guidance, hypotension occurred in 6 patients in a group receiving propofol, as compared to 12 patients receiving propofol/remifentanil intravenous analgesia, with no statistical significance related to HR and MAP values. 

An anesthesia modality should guarantee patients undergoing CP safety and comfort and enable their fast recovery and discharge [44]. In the current study, no statistically significant differences were found in terms of patients’ or endoscopists’ satisfaction, regardless of the group allocation. Therefore, the utility of extra costly monitoring, especially AoA, in view of the abovementioned finding does not seem to be justified, as only three patients and five endoscopists out of a total of 158 cases included in the final analysis, regardless of the group allocation, declared dissatisfaction with the performed ISA. Similarly, Soral et al. [33], in the abovementioned study with ANI vs. conventional methods in patients undergoing CPs, observed no difference in NRS scores intraoperatively, as compared to conventional methods [33]. Similarly, no significant difference was found between patients’ and endoscopist’ satisfaction by Bilgi et al. [43], who compared the effects of the administration of propofol alone and the administration of remifentanil in addition to propofol on patients’ and endoscopists’ satisfaction under BIS guidance with a target value of 70–75 [43]. 

The employment of AoA monitoring was reported to lead to a reduction in the use of propofol, as compared to standard practice in patients undergoing propofol/remifentanil anesthesia [24]. Therefore, in the current study, the economizing of resources was studied, as a somewhat similar anesthetic modality was used. Statistically significantly more propofol consumption was found for SE when monitoring the depth of sedation in both the SE and AoA groups, compared to the control group. Sargin et al. [45] aimed to evaluate the effect of BIS monitoring on early cognitive performance among patients undergoing sedation for CPs. Contrary to the current study findings, they observed that BIS monitoring among sedated patients was associated with lower propofol use and a smaller decline in cognitive performance, as compared to standard practice [45]. The same conclusion was drawn by Bellolio et al. [41], who observed that BIS monitoring among sedated patients was associated with lower propofol use compared to the control group [41]. 

In the current study, the employment of SPI to guide the titration of FNT led to a statistically significant increase in FNT consumption, as compared to both the SE and control groups. Nevertheless, the increased consumption of FNT in the AoA group as compared to the SE group did not lead to a decrease in the rate of IPP, so its utility had no clinical relevance and imposed an unnecessary cost. A different conclusion was drawn by Won et al. [40], who reported a reduced opioid consumption in patients for whom SPI was utilized to guide opioid titration, as compared to standard practice. 

Our study has several limitations. First, the time of emergence from ISA was not evaluated because different anesthetic modalities were used. In Groups SE and AoA, the patient was referred to PACU after the completion of the CP, when SE was equal to the SE value during Stage 1, whereas, in the control group, the patient was referred after eye opening and verbal response. Second, the CP was performed by several different endoscopists, all the patients were prepared for the CP, and they were performed according to the same algorithm of the Polish Society of Gastroenterology under the professional supervision of the Head of the Unit of Endoscopy by the Department of Gastroenterology (J.E.). Moreover, the number of patients allocated to each group—over 50—enabled the equal participation of every endoscopist in the performance of the procedure and its completion in each studied group. Third, pain perception is a subjective phenomenon; despite preoperative training regarding the use of NRS, patients may not be able to define their perception of pain using a scale and report it consistently [46]. In addition, in the course of ISA, no muscular blockade is used. Thus, visceral nociceptive afferent stimulation during CP, especially when accompanied by insufficient analgesia, may result in an increase in RE and result from an increase in SE score, indicating a false depth of sedation, which can be misinterpreted by an anesthesiologist as patient arousal and can lead to wrongful decisions about the administration of HD [41,47]. Finally, the current literature provides no consistent algorithm for the titration of rescue opioid analgesics based on the observance of SPI value fluctuations online [35]. Therefore, similar to our previous study, we adopted a methodology where the intraprocedural increase of SPI value by 15% constituted an indication to administer a single rescue dose of intravenous FNT [21].

## 4. Materials and Methods

### 4.1. Patients

The protocol of the study was in compliance with the 1964 Helsinki Declaration and was approved by the Local Bioethics Committee at the Medical University of Silesia in Katowice, Poland (approval number: KNW/0022/KB/141/18). The study was registered in the Clinical Trials Registry (ID: NCT03922815). Patients scheduled for a CP under ISA in the Unit of Endoscopy at Department of Gastroenterology at the 5th Regional Hospital between September 2019 and January 2020 were the subjects of the study. Randomization was performed by the principal investigator (M.S.) by opening sealed envelopes after obtaining written informed consent.

One hundred and sixty-six patients aged 18–75 years, with American Society of Anesthesiologists (ASA) Physical Status I–III, were recruited and randomly allocated to one of the following three groups for CPs: (1) patients whose analgesic drugs were given using a conventional method, and the depth of sedation was monitored with Entropy SE (the SE group); (2) patients whose analgesic drugs were guided by SPI monitoring, and the depth of sedation was monitored with Entropy SE (the Adequacy of Anesthesia (AoA) group); or (3) patients whose anesthetic drugs were given using a conventional techniques based on the anesthesiologists’ intuition accompanied by observation of hemodynamic values’ fluctuations, together with the absence of ciliary reflex and face grimaces, regarding the rate of selected adverse events in the perioperative period (the control group). 

Sample size (for estimating the proportion of population) was estimated at 166, with a confidence level of 95% and a margin of error of 5%, and the expected proportion of patients with pain with a Numeric Pain Rating Scale (NRS) score of >3 in preliminary examinations was 12.3% (*n* = 50).

Exclusion criteria were dysmorphic facial features making entropy monitoring impossible, unanticipated complications during the procedure that, in the opinion of the investigators, may have influenced the results, heart arrhythmias, a pacemaker in situ, requisite intraprocedural vasoactive drugs administration, chronic pain treatment and chronic pain itself, drug allergies, a history of opioid drug abuse, and a refusal to continue to participate in the study. 

### 4.2. Anesthetic Technique

Prior to the CP, patients were informed about the possibility of IPP, PPP, and PONV. They were trained on the use of the Numeric Pain Rating Scale (NRS) to report pain. On a scale from 0 to 10, 0 indicated no pain, and 10 indicated the worst pain imaginable. Patients were also evaluated for the risk of PONV using the APFEL score before the procedure in order to ensure the homogeneity of the groups. Apfel score is a widely recognized risk score having broad applicability in predicting the incidence of PONV in adults undergoing anesthesia for various types of surgical procedures. Female gender, history of motion sickness or PONV, nonsmoking status, and the use of postoperative opioids were recognized to constitute the independent risk factors of occurrence of PONV. If none, one, two, three, or four of these risk factors were present, the incidences of PONV were estimated as follows: 10%, 21%, 39%, 61%, and 79%, respectively [48,49].

Prior to the induction of ISA, 10 mL/kg of Optylite Solution (Fresenius Kabi, Kutno, Poland) was administered intravenously. At the induction of ISA, performed according to the current guidelines of the ASA Committee on Quality Management and Departmental Administration regarding the definition of general anesthesia and the levels of sedation/analgesia (43), patients in all groups received a bolus of 1 mcg/kg of FNT (Fentanyl WZF, 50 mcg/mL; 2m, Polfa Warsawa, S.A, Warsaw, Poland) intravenously, and all patients were subsequently induced with 0.5 mg/kg of propofol every 2 min until the depth of sedation reached SE < 70 in the SE and AoA groups and until the eyelash reflex was lost in the control group. During maintenance, additional boluses of 0.5 mg/kg of propofol were administered intravenously in the SE and AoA groups with a target value of 60–70 and using conventional methods in the control group. In the event of inadequate analgesia, confirmed by an increase in SPI >15 from baseline values in the AoA group or an increase in mean blood pressure and heart rate >30% from baseline values in the SE and control groups, additional boluses of 0.5 mcg/kg of FNT were given intravenously at 2-min intervals until a normalization of SPI values in the AoA group or a normalization of hemodynamic parameters in the SE and control group. 

Intraprocedural monitoring included pulse oximetry (SpO_2_), electrocardiography (ECG), non-invasive blood pressure monitoring of systolic arterial pressure (SAP), mean arterial pressure (MAP), diastolic arterial pressure (DAP), RE and SE, and SPI, according to group allocation. A single dose of 10 mg of ephedrine (Ephedrinum hydrochloricum WZF 25 mg/mL; 1 mL Polfa Warsawa, Poland) was administered if MAP decreased <60 mmHg, and a single dose of 0.5 mg of atropine (Atropinum sulfuricum WZF 1 mg/mL; 1 mL Polfa Warsawa, Poland) was administered if HR decreased <45 bpm, at 3 min intervals, until the abovementioned values returned to the normal level. 

Postoperatively, patients were evaluated during the first 24 h for incidence of PONV and pain intensity using the NRS scale, and patients’ and endoscopists’ satisfaction was graded using a specially designed satisfaction scale. In the case of PPP, to meet each patient’s specific needs, postoperative analgesia was provided according to the current guidelines of the Polish Society of Anesthesiology and Intensive Therapy [30]. 

The statistical analysis perioperative period was divided into three stages: Stage 1: before the induction of ISA; Stage 2: CP; Stage 3: observance after the CP in a postanesthesia care unit (PACU) until full patient recovery from ISA. 

### 4.3. Colonoscopy Technique

According to the guidelines, all patients were prepared for the CP [50], which was performed by a team of three endoscopists with over 10 years of experience in CP performance to ensure the homogeneity of CP techniques [44], which is crucial in terms of the influence of intraprocedural visceral nociceptive stimulation on the vasoactive reaction expressed by either haemodynamic values or SPI changes. 

### 4.4. Evaluation of Patients and Endoscopist Satisfaction 

The satisfaction of patients and endoscopists was assessed on the basis of a proprietary questionnaire that the recipients were asked to fill in.

Endoscopists were asked if they were satisfied with the sedation for the colonoscopy procedure, taking into account factors such as depth of sedation, movement of the patient during procedure, and suboptimal analgesia. They could express their satisfaction as (1) not satisfied, (2) moderately satisfied, (3) satisfied, or (4) very satisfied.

In turn, the patients were asked if they felt pain during and after the colonoscopy procedure and, if so, how bad the pain was on a 0–10 scale. In addition, patients were asked to rate their satisfaction with the pain management during the colonoscopy procedure on a scale of 1–4: (1) not satisfied, (2) moderately satisfied, (3) satisfied, or (4) very satisfied.

### 4.5. Statistical Analysis

The sample size was estimated at 166, with a confidence level of 95% and a margin of error of 5%, and the expected proportions of patients with pain measured with a Numeric Pain Rating Scale (NRS) score of >3 in preliminary examinations was 12.3% (*n* = 50).

Statistical calculations were performed using MS Excel, STATISTICA 13.3, Stat Soft, Cracow, Poland. The measured data were characterized using the mean and standard deviation—X ± SD—and the median with interquartile range—M (IQR). Normality of distribution was checked with the Shapiro–Wilk W test. The significance of differences between means was tested using ANOVA for multiple groups; for skewed distributions, the compatibility of groups was examined using the Kruskal–Wallis test by ranks. For nominal data, we used percentages. The significance of differences between percentages was tested using the test for two proportions. Relationships between nominal variables were verified by the X^2^ test of independence. Statistical significance was set to *p* < 0.05.

## 5. Conclusions

In patients undergoing a CP under ISA using propofol and FNT, as compared to the standard practice, intraprocedural SE monitoring was crucial to reduce the rate of IAwR and IPP, with no influence on the rate of PPP and PONV or patients’ and endoscopists’ satisfaction. Further employment of AoA guidance for propofol and FNT titration, as compared to SE monitoring only, did not reduce the occurrence of the aforementioned studied parameters in patients undergoing CPs under ISA and therefore imposed unnecessary costs.

## Figures and Tables

**Table 1:** Comparison of values of HR, SAP, MAP, and DAP in patients before the induction of ISA between the control, SE, and AoA groups.

Parameter	Total *n =* 158 (100%)	Group C *n =* 53 (33.5%)	Group SE *n =* 52 (32.9%)	Group AoA *n =* 53 (33.5%)	*p*-Value
Stage 1—ONSET					
HR (beats/min)	76.4 ± 14.3 73 (20)	76.1 ± 12.8 75 (20)	77.4 ± 16 72 (22)	75.6 ± 14.1 71 (19)	*p* = 0.91
SAP (mmHg)	137.2 ± 22.3 133.5 (27)	138.1 ± 23.1 133 (25)	139.8 ± 20.5 138 (26.5)	133.8 ± 23.1 132 (30)	*p* = 0.23
MAP (mmHg)	98.3 ± 13.9 97.5 (18)	98.2 ± 13.2 97 (13)	100.2 ± 14.1 99.5 (17)	96.6 ± 14.2 96 (18)	*p* = 0.34
DAP (mmHg)	70 ± 11.7 68.5 (16)	69.1 ± 10.8 68 (15)	71.3 ± 12.7 69 (16.5)	69.6 ± 11.6 68 (15)	*p* = 0.68
SE	87.3 ± 4.9 89 (2)	-	87.7 ± 3.5 89 (2)	87 ± 6 89 (2)	*p* = 0.56
SPI	60.4 ± 20.7 68.5 (32)	-		60.1 ± 20.7 68 (28)	-
Parameter	Total *n =* 158 (100%)	Group C *n =* 53 (33.5%)	Group SE *n =* 52 (32.9%)	Group AoA *n =* 53 (33.5%)	*p*-value
Stage 2—CP					
mean HR (beats/min)	67 ± 9.5 65.5 (13.3)	68.2 ± 10.2 65.7 (14.5)	65.3 ± 9.1 64.9 (12.5)	67.3 ± 9.1 64.7 (12.7)	*p* = 0.47
mean SAP (mmHg)	114.8 ± 23.2 111.5 (30.1)	124.4 ± 23.5 124 (35.8)	112.2 ± 24.4 111.3 (31.5)	107.6 ± 18.2 107 (26)	*p* = 0.0004 *p* < 0.001 ^A,B^
mean MAP (mmHg)	84.1 ± 14.8 82.8 (21.1)	89.5 ± 14.6 89.5 (21.4)	82.9 ± 15.7 82 (21.7)	79.9 ± 12.5 80 (16.4)	*p* = 0.002 *p* < 0.01 ^C^
mean DAP (mmHg)	61.6 ± 11.6 61.2 (16.6)	64.6 ± 11.3 66 (16)	61 ± 12.5 59 (15.8)	59.3 ± 10.3 59 (14)	*p* = 0.06
mean SE	66.3 ± 9.6 69.2 (10.3)	-	67.4 ± 8 68.8 (9.7)	65.3 ± 10.8 69.5 (13.4)	*p* = 0.69
mean SPI	36.4 ± 14.7 33.1 (16.7)	-	-	36.4 ± 14.7 33.1 (16.7)	-
Parameter	Total *n =* 158 (100%)	Group C *n =* 53 (33.5%)	Group SE *n =* 52 (32.9%)	Group AoA *n =* 53 (33.5%)	*p*-value
Stage 3—PACU					
mean HR (beats/min)	67.9 ± 9.7 67.5 (14.3)	69.3 ± 10.5 70 (17)	67.5 ± 9.4 66.4 (12.3)	66.9 ± 9.3 67.6 (14.3)	*p* = 0.47
mean SAP (mmHg)	113.7 ± 21.3 111 (28.8)	119.7 ± 24.8 118.8 (33)	114.2 ± 20.1 113.8 (24)	107.2 ± 16.5 104.6 (22)	*p* = 0.01 *p* < 0.05 ^C^
mean MAP (mmHg)	83.2 ± 14 81.8 (20)	86.5 ± 16.1 89 (24.3)	83.5 ± 13.3 82.2 (17.3)	79.6 ± 11.7 78 (16.2)	*p* = 0.04 *p* < 0.05 ^D^
mean DAP (mmHg)	61.6 ± 12 61.2 (17.3)	63.5 ± 12.3 64 (18.8)	61.3 ± 11.5 60.8 (15.6)	60.1 ± 12.1 58.3 (14.2)	*p* = 0.21
mean SE	83.6 ± 6.7 85.3 (6.1)	-	83.5 ± 7.5 85.5 (6.7)	83.8 ± 4.5 84.4 (6)	*p* = 0.54
mean SPI	38 ± 14 33.4 (16.4)	-	-	38 ± 14 33.4 (16.4)	-

Results are presented as mean ± SD and median (IQR) for quantitative variables. *p*-values were found by a one-way ANOVA test for quantitative variables. *p*-values were found by post hoc tests. ^A^: Significantly less in Group SE than in Group C (*p* < 0.05). ^B^: Significantly less in Group AoA than in Group C (*p* < 0.001). ^C^: Significantly less in Group AoA than in Group C (*p* < 0.01)). ^D^: Significantly less in Group AoA than in Group C (*p* < 0.05). Abbreviations: Group C: control group; Group SE: Entropy SE group; Group AoA: Adequacy of Anesthesia group; HR: heart rate; SAP: systolic arterial pressure; MAP: mean arterial pressure; DAP: diastolic arterial pressure; SE: state entropy; SPI: surgical pleth index; SD: standard deviation; IQR: interquartile range.

**Table 2:** Comparison of optimalization of resources and the necessity of rescue atropine and ephedrine administration in patients according to allocation to the studied groups.

Parameter	Total *n =* 158 (100%)	Group C *n =* 53 (33.5%)	Group SE *n =* 52 (32.9%)	Group AoA *n =* 53 (33.5%)	*p*-Value
Intraprocedural rescue FNT consumption (microgram)	102.8 ± 26.2 100 (0)	98.1 ± 21.3 100 (0)	90.4 ± 23.8 100 (12.5)	119.8 ± 24.2 100 (50)	*p* < 0.0001 ^A,B^
Intraprocedural propofol consumption (mg)	156.7 ± 64.3 150 (80)	128.9 ± 57.5 120 (70)	179.6 ± 62.2 180 (75)	162.2 ± 63.5 160 (80)	*p* < 0.0001 ^A,C^
Intraprocedural requirement for intravenous crystalloids (mL)	493.7 ± 79.6 500 (0)	471.7 ± 116.6 500 (0)	509.6 ± 69.3 500 (0)	500 ± 0 500 (0)	*p* = 0.36
Length of CP (min)	11.9 ± 6.9 10 (8)	11.4 ± 7.6 9 (8)	11.6 ± 6 10 (8)	12.6 ± 6.9 11 (7)	*p* = 0.37
Necessity of intraprocedural rescue atropine administration (number of patients)	12 (7.6%)	6 (11.3%)	5 (9.6%)	1 (1.9%)	*p* = 0.1
Necessity of intraprocedural rescue ephedrine administration (number of patients)	2 (1.3%)	0 (0%)	1 (1.9%)	1 (1.9%)	*p* = 0.44

Results are presented as a mean ± SD and median (IQR) for quantitative variables and numbers (percentages) for nominal variables. *p*-values were found by a one-way ANOVA test for quantitative variables; *p*-values were found by an X^2^ test for nominal variables. *p*-values were found by post hoc tests. ^A^: Significantly more in Group AoA than in Group C (*p* < 0.01). ^B^: Significantly more in Group AoA than in Group SE (*p* < 0.001). ^C^: Significantly more in Group SE than in Group C (*p* < 0.001). Abbreviations: Group C: control group; Group SE: Entropy SE group; Group AoA: Adequacy of Anesthesia group; SD: standard deviation; IQR: interquartile range.

**Table 3:** Survey results of the patients and endoscopist satisfaction.

Survey	Total *n =* 158 (100%)	Group C *n =* 53 (33.5%)	Group SE *n =* 52 (32.9%)	Group AoA *n =* 53 (33.5%)	*p*-Value
APFEL score	1.44 ± 0.77 1 (1)	1.45 ± 0.82 2 (1)	1.46 ± 0.8 1 (1)	1.4 ± 0.69 1 (1)	*p* = 0.89
Apfel (%)	0.3 ± 0.13 0.21 (0.18)	0.3 ± 0.14 0.39 (0.18)	0.3 ± 0.15 0.21 (0.18)	0.29 ± 0.12 0.21 (0.18)	*p* = 0.89
PONV No/Yes	155 (98.1%)/ 3 (1.9%)	51 (96.2%)/ 2 (3.8%)	52 (100%)/ 0 (0%)	52 (98.1%)/ 1 (1.9%)	*p* = 0.25
Number of patients declaring unacceptable PPP (NRS > 3)	20 (12.7%)	8 (15.1%)	7 (13.5%)	5 (9.4%)	*p* = 0.66
Number of patients declaring acceptable PPP (NRS < 4)	138 (87.3%)	45 (84.9%)	45 (86.5%)	48 (90.6%)	*p* = 0.66
NRS at admission to PACU	0.7 ± 1.6 0 (0)	1.1 ± 1.9 0 (2)	0.7 ± 1.6 0 (0)	0.2 ± 0.6 0 (0)	*p* = 0.08
NRS 2 at discharge from PACU	0.86 ± 1.7 0 (0)	1 ± 1.9 0 (1)	0.8 ± 1.6 0 (0)	0.8 ± 1.6 0 (0)	*p* = 0.85
Endoscopists’ satisfaction with quality of performed ISA (no/yes)	5 (3.2%) / 153 (96.8%)	3 (5.7%)/ 50 (94.3%)	2 (3.8%)/ 50 (96.2%)	0 (0%)/ 53 (100%)	*p* = 0.11
Level of endoscopists’ satisfaction with quality of performed ISA using Likert scale (0–4)	3.7 ± 0.5 4 (0)	3.7 ± 0.6 4 (0)	3.6 ± 0.7 4 (1)	3.9 ± 0.3 4 (0)	*p* = 0.99
Patient satisfaction with quality of performed ISA (no/yes)	3 (1.9%)/ 155 (98.1%)	2 (3.8%)/ 51 (96.2%)	1 (1.9%)/ 51 (98.1%)	0 (0%)/ 53 (100%)	*p* = 0.24
Level of patient satisfaction with quality of performed ISA using Likert scale (0–4)	3.9 ± 0.3 4 (0)	3.8 ± 0.5 4 (0)	3.9 ± 0.3 4 (0)	4 ± 0.1 4 (0)	*p* = 0.09
Number of patients declaring intraprocedural awareness with recall (IAwR)	24 (15.3%)	15 (28.8%)	7 (13.5%)	2 (3.8%)	*p* = 0.001 *p* < 0.01 ^A^
Number of patients declaring intraprocedural (IPP) (NRS > 3)	9 (5.7%)	7 (13.2%)	1 (1.9%)	1 (1.9%)	*p* = 0.02 *p* < 0.05 ^A^
Value of IPP using NRS	0.3 ± 1.3 0 (0)	0.8 ± 2 0 (0)	0.1 ± 0.7 0 (0)	0 ± 0.3 0 (0)	*p* = 0.0003 *p* < 0.001 ^A^

Results are presented as a mean ± SD and median (IQR) for quantitative variables and numbers (percentages) for nominal variables. *p*-values were found by a one-way ANOVA test for quantitative variables; *p*-values were found by an X^2^ test for nominal variables. *p*-values were found by post hoc tests. ^A^: Significantly more in Group C than in Groups SE and AoA (*p* < 0.001, both). Abbreviations: Group C: control group; Group SE: Entropy SE group; Group AoA: Adequacy of Anesthesia group; NRS: Numeric Pain Rating Scale; SD: standard deviation; IQR: interquartile range.

**Table 4:** Comparison of maximum and minimum values of monitored parameters of patients between the studied groups during the CP.

Parameter	Total *n =* 158 (100%)	Group C *n =* 53 (33.5%)	Group SE *n =* 52 (32.9%)	Group AoA *n =* 53 (33.5%)	*p*-Value
Stage 2—CP					
max HR (beats/min)	76 ± 12.8 74 (15)	77.6 ± 12.7 75 (20)	73.3 ± 11.2 71.5 (12)	77.1 ± 14 75 (16)	*p* = 0.19
max SAP (mmHg)	124.5 ± 26.7 119.5 (31)	134.3 ± 27.2 128 (44)	121.3 ± 27.3 116 (32.5)	117.8 ± 23 115 (25)	*p* = 0.004 *p* < 0.01 ^A,B^
max MAP (mmHg)	90.9 ± 17.3 88.5 (22)	95.9 ± 17.6 95 (21)	89.5 ± 18.6 87 (26)	87.2 ± 14.7 87 (20)	*p* = 0.03 *p* < 0.05 ^C^
max DAP (mmHg)	67 ± 13.3 65 (17)	69.2 ± 13.4 69 (16)	66.6 ± 14.3 65 (14.5)	65.2 ± 12 62 (19)	*p* = 0.32
max SE	85 ± 7.3 87 (6)	-	84.3 ± 7.4 87 (7.5)	85.7 ± 7.2 88 (4)	*p* = 0.14
max SPI	53.1 ± 17.7 53 (26)	-	-	53.1 ± 17.7 53 (26)	-
min HR (beats/min)	60.4 ± 9.3 59 (13)	60.4 ± 10.6 60 (15)	59.2 ± 8.7 58 (13)	61.6 ± 8.3 59 (10)	*p* = 0.42
min SAP (mmHg)	105.6 ± 21.6 105 (27)	114.3 ± 22.8 116 (28)	104.5 ± 21.7 107 (22)	98.1 ± 17.2 100 (20)	*p* = 0.0004 *p* < 0.001 ^D^
min MAP (mmHg)	77.4 ± 14.4 79 (19)	82.4 ± 14.9 82 (22)	76.3 ± 14.5 78.5 (17)	73.6 ± 12.4 74 (14)	*p* = 0.005 *p* < 0.01 ^A^
min DAP (mmHg)	56.5 ± 11.7 56 (15)	60.1 ± 11.7 60 (19)	55.4 ± 12.4 56 (13.5)	53.9 ± 10.3 54 (13)	*p* = 0.02 *p* < 0.05 ^C^
min SE	45.7 ± 14.2 48 (21)		45.6 ± 12.6 48.5 (18)	45.5 ± 15.5 47 (24)	*p* = 0.97
min SPI	23.8 ± 12.8 20 (12)	-	-	23.8 ± 12.8 20 (12)	-
Stage 3 –PACU					
max HR (beats/min)	72.3 ± 11.6 71 (18)	71.7 ± 12.7 70 (19.5)	72.6 ± 10.3 71 (11)	72.5 ± 11.8 71 (22)	*p* = 0.93
max SAP (mmHg)	119.5 ± 22.8 117 (29)	123.8 ± 27 121 (39)	121.1 ± 22.6 119.5 (28)	113.6 ± 16.9 112 (27)	*p* = 0.12
max MAP (mmHg)	87.3 ± 15.2 86 (20)	89.2 ± 18.2 90 (26)	88.1 ± 14.7 84.5 (19.5)	84.6 ± 12 85 (20)	*p* = 0.34
max DAP (mmHg)	65.2 ± 12.4 65 (18)	65.7 ± 13.9 67 (21)	66.3 ± 12.3 66 (19)	63.7 ± 10.8 64 (13)	*p* = 0.56
max SE	88.6 ± 5.8 90 (1)	-	89.3 ± 2.7 90 (2)	88 ± 7.7 90 (1)	*p* = 0.46
max SPI	52 ± 16.1 48 (25.5)	-	-	52 ± 16.1 48 (25.5)	-
min HR (beats/min)	63.4 ± 9.5 62 (12)	64.5 ± 10.3 65.5 (16)	63.5 ± 9.4 62 (12)	62.3 ± 8.7 61 (10)	*p* = 0.51
min SAP (mmHg)	106.9 ± 21.3 105 (29)	113.8 ± 25.2 115.5 (35)	106.4 ± 20.2 103.5 (24)	100.7 ± 15.8 100 (17)	*p* = 0.009 *p* < 0.01 ^A^
min MAP (mmHg)	78.1 ± 13.9 77 (20)	82.6 ± 15.8 83.5 (22.5)	77.6 ± 13.6 77 (17.5)	74.3 ± 11.1 72 (15)	*p* = 0.01 *p* < 0.05 ^C^
min DAP (mmHg)	56.9 ± 11.6 56 (16)	59.8 ± 12.1 60.5 (19.4)	56.4 ± 12.2 56 (16)	54.5 ± 10 53 (15)	*p* = 0.06
min SPI	27 ± 14.9 23 (14)	-	-	25.1 ± 11.2 23 (10)	-

Results are presented as a mean ± SD and median (IQR) for quantitative variables. *p*-values were found by a one-way ANOVA test for quantitative variables. *p*-values were found by post hoc tests. ^A^: Significantly less in Group AoA than in Group C (*p* < 0.01). ^B^: Significantly less in Group SE than in Group C (*p* < 0.05). ^C^: Significantly less in Group AoA than in Group C (*p* < 0.05). ^D^: Significantly less in Group AoA than in Group C (*p* < 0.001). Abbreviations: Group C: control group; Group SE: Entropy SE group; Group AoA: Adequacy of Anesthesia group; HR: heart rate; SAP: systolic arterial pressure; MAP: mean arterial pressure; DAP: diastolic arterial pressure; SPI: surgical pleth index; SD: standard deviation; IQR: interquartile range.

**Table 5:** Changes in minimum and maximum SPI values with z hemodynamic values in the AoA group.

Correlation	Total *n* (100%)	Yes	No	% Yes/No	*p*-Value
SPI max vs. HR max	48 (90.6%)	26 (49.1%)	22 (41.5%)	118.2%	*p* = 0.43
SPI max vs. MAP max	41 (77.4%)	15 (28.3%)	26 (49.1%)	57.7%	*p* = 0.03 *p* < 0.05 ^A^
SPI min vs. HR min	50 (94.3%)	23 (43.4%)	27 (50.9%)	85.2%	*p* = 0.44
SPI min vs. MAP min	31 (58.5%)	14 (26.4%)	17 (32.1%)	82.4%	*p* = 0.52

Results are presented as numbers (percentages) for nominal variables. *p*-values were found by test for two proportions. ^A^: Significantly more without correlation (*p* < 0.05). Abbreviations: SPI: surgical pleth index; HR: heart rate; MAP: mean arterial pressure.

## Data Availability

The data used to support the findings of this study are included in the article. The data will not be shared due to third-party rights and commercial confidentiality.

## Supplementary Materials

The following are available online at https://www.mdpi.com/article/10.3390/ph14050464/s1. Figure S1. Randomization graph—study outline. Table S1. Anthropometric data of patients in the studied groups: the control group, the SE group, and the AoA group.

## References

[1] Erstad D.J., Krowsoski L.S., Kaafarani H.M. (2017). Abdominal Pain after Colonoscopy. Gastroenterology.

[2] Baker F.A., Mari A., Aamarney K., Hakeem A.R., Ovadia B., Kopelman Y. (2019). Propofol sedation in colonoscopy: From satisfied patients to improved quality indicators. Clin. Exp. Gastroenterol..

[3] Early D.S., Lightdale J.R., Vargo J.J., Acosta R.D., Chandrasekhara V., Chathadi K.V., DeWitt J.M. (2018). Guidelines for sedation and anesthesia in GI endoscopy. Gastrointest. Endosc..

[4] Pace D., Borgaonkar M. (2018). Deep sedation for colonoscopy is unnecessary and wasteful. CMAJ.

[5] Gruenewald M., Ilies C. (2013). Monitoring the nociception–anti-nociception balance. Best Pract. Res. Clin. Anaesthesiol..

[6] Maja V., Talja P., Tenkanen N., Tolvanen-Laakso H. (2004). Description of the Entropy™ algorithm as applied in the datex-ohmeda S/5™ entropy module. Acta Anaesthesiol. Scand..

[7] Gruenewald M., Zhou J., Schloemerkemper N., Meybohm P., Weiler N., Tonner P.H., Bein B. (2007). M-Entropy guidance vs. standard practice during propofol-remifentanil anaesthesia: A randomised controlled trial. Anaesthesia.

[8] Horn C.C., Wallisch W.J., Homanics G.E., Williams J.P. (2014). Pathophysiological and neurochemical mecha-nisms of postoperative nausea and vomiting. Eur. J. Pharmacol..

[9] Yoldas H., Yildiz I., Karagoz I., Sit M., Ogun M.N., Demirhan A., Bilgi M. (2019). Effects of Bispectral Index-controlled Use of Magnesium on Propofol Consumption and Sedation Level in Patients Undergoing Colonoscopy. Medeni. Med. J..

[10] Ferreira A.O., Torres J., Barjas E., Nunes J., Glória L., Ferreira R., Cravo M. (2016). Non-anesthesiologist administration of propofol sedation for colonoscopy is safe in low risk patients: Results of a noninferiority randomized controlled trial. Endoscopy.

[11] Becerra L., Aasted C.M., Boas D.A., George E., Yücel M.A., Kussman B.D., Borsook D. (2016). Brain measures of nociception using near infrared spectroscopy in patients undergoing routine screening colonoscopy. Pain.

[12] Lee H., Kim J.H. (2009). Superiority of split dose midazolam as conscious sedation for outpatient colonoscopy. World J. Gastroenterol. WJG.

[13] Forster C., Vanhaudenhuyse A., Gast P., Louis E., Hick G., Brichant J.F., Joris J. (2018). Intravenous infusion of lidocaine significantly reduces propofol dose for colonoscopy: A randomised placebo-controlled study. Br. J. Anaesth..

[14] Stasiowski M.J., Duława A., Król S., Marciniak R., Kaspera W., Niewiadomska E., Jałowiecki P. (2020). Polyspikes and Rhythmic Polyspikes during Volatile Induction of General Anesthesia with Sevoflurane Result in Bispectral Index Variations. Clin. EEG Neurosci..

[15] Stasiowski M., Duława A., Szumera I., Marciniak R., Niewiadomska E., Kaspera W., Jałowiecki P. (2020). Variations in Values of State, Response Entropy and Haemodynamic Parameters Associated with Development of Different Epileptiform Patterns during Volatile Induction of General Anaesthesia with Two Different Anaesthetic Regimens Using Sevoflurane in Comparison with Intravenous Induct: A Comparative Study. Brain Sci..

[16] Stasiowski M.J., Marciniak R., Duława A., Krawczyk L., Jałowiecki P. (2019). Epileptiform EEG patterns during different techniques of induction of general anaesthesia with sevoflurane and propofol: A randomised trial. Anaesthesiol. Intensive Ther..

[17] Musialowicz T., Lahtinen P. (2014). Current Status of EEG-Based Depth-of-Consciousness Monitoring During General Anesthesia. Curr. Anesthesiol. Rep..

[18] Gruenewald M., Willms S., Broch O., Kott M., Steinfath M., Bein B. (2014). Sufentanil administration guided by surgical pleth index vs. standard practice during sevoflurane anaesthesia: A randomized controlled pilot study. Br. J. Anaesth..

[19] Gruenewald M., Meybohm P., Ilies C., Höcker J., Hanss R., Scholz J., Bein B. (2009). Influence of different remifentanil concentrations on the performance of the surgical stress index to detect a standardized painful stimulus during sevoflurane anaesthesia. Br. J. Anaesth..

[20] Bonhomme V., Uutela K., Hans G., Maquoi I., Born J.D., Brichant J.F., Hans P. (2011). Comparison of the Surgical Pleth Index™ with haemodynamic variables to assess nociception–anti-nociception balance during general anaesthesia. Br. J. Anaesth..

[21] Stasiowski M., Missir A., Pluta A., Szumera I., Stasiak M., Szopa W., Kaspera W. (2020). Influence of infiltration anaesthesia on perioperative outcomes following lumbar discec-tomy under surgical pleth index-guided general anaesthesia: A preliminary report from a randomised controlled prospective trial. Adv. Med. Sci..

[22] Fratino S., Peluso L., Talamonti M., Menozzi M., Costa Hirai L.A., Lobo F.A., Prezioso C., Creteur J., Payen J., Taccone F.S. (2021). Evaluation of Nociception Using Quantitative Pupillometry and Skin Conductance in Critically Ill Unconscious Patients: A Pilot Study. Brain Sci..

[23] Chen X., Thee C., Gruenewald M., Wnent J., Illies C., Hoecker J., Bein B. (2010). Comparison of surgical stress index-guided analgesia with standard clinical practice during routine general anesthesia: A pilot study. Anesthesiology.

[24] Kim J.H., Jwa E.K., Choung Y., Yeon H.J., Kim S.Y., Kim E. (2020). Comparison of Pupillometry with Surgical Pleth Index Monitoring on Perioperative Opioid Consumption and Nociception during Propofol-Remifentanil Anesthesia: A Prospective Randomized Controlled Trial. Anesth. Analg..

[25] Gruenewald M., Harju J., Preckel B., Molnár Z., Yli-Hankala A., Rosskopf F., AoA Study Group (2021). Comparison of adequacy of anaesthesia monitoring with standard clinical practice monitoring during routine general anaesthesia: An international, multicentre, single-blinded randomised controlled trial. Eur. J. Anaesthesiol..

[26] Stasiowski M.J., Pluta A., Lyssek-Boroń A., Kawka M., Krawczyk L., Niewiadomska E., Grabarek B.O. (2021). Preventive Analgesia, Hemodynamic Stability, and Pain in Vitreoretinal Surgery. Medicina.

[27] Stogiannou D., Protopapas A., Protopapas A., Tziomalos K. (2018). Is propofol the optimal sedative in gastrointestinal endoscopy?. Acta Gastroenterol. Belg..

[28] Musialowicz T., Valtola A., Hippeläinen M., Halonen J., Lahtinen P. (2017). Spectral Entropy Parameters during Rapid Ventricular Pacing for Transcatheter Aortic Valve Implantation. Entropy.

[29] Musialowicz T., Lahtinen P., Pitkänen O., Kurola J., Parviainen I. (2011). Comparison of Spectral Entropy and BIS VISTA™ monitor during general anesthesia for cardiac surgery. J. Clin. Monit. Comput..

[30] Yli-Hankala A. (1990). The effect of nitrous oxide on EEG spectral power during halothane and isoflurane anaesthesia. Acta Anaesthesiol. Scand..

[31] Jameson L.C., Sloan T.B. (2006). Using EEG to monitor anesthesia drug effects during surgery. J. Clin. Monit. Comput..

[32] Chhabra A., Subramaniam R., Srivastava A., Prabhakar H., Kalaivani M., Paranjape S. (2016). Spectral entropy monitoring for adults and children undergoing general anaesthesia. Cochrane Database Syst. Rev..

[33] Soral M., Altun G.T., Dinçer P.Ç., Arslantaş M.K., Aykaç Z. (2020). Effectiveness of the analgesia nociception index monitoring in patients who undergo colonoscopy with sedo-analgesia. Turk. J. Anaesthesiol. Reanim..

[34] Ledowski T., Pascoe E., Ang B., Schmarbeck T., Clarke M.W., Fuller C., Kapoor V. (2010). Monitoring of intra-operative nociception: Skin conductance and surgical stress index versus stress hormone plasma levels. Anaesthesia.

[35] Ledowski T., Burke J., Hruby J. (2016). Surgical pleth index: Prediction of postoperative pain and influence of arousal. Br. J. Anaesth..

[36] Zhang W., Zhu Z., Zheng Y. (2018). Effect and safety of propofol for sedation during colonoscopy: A meta-analysis. J. Clin. Anesth..

[37] Vanluchene A.L.G., Struys M.M.R.F., Heyse B.E.K., Mortier E.P. (2004). Spectral entropy measurement of patient responsiveness during propofol and remifentanil. A comparison with the bispectral index. Br. J. Anaesth..

[38] Seleem W.M., El Hossieny K.M., Abd-Elsalam S. (2020). Evaluation of different sedatives for colonoscopy. Curr. Drug Saf..

[39] Bergmann I., Göhner A., Crozier T.A., Hesjedal B., Wiese C.H., Popov A.F., Hinz J.M. (2013). Surgical pleth index-guided remifentanil administration reduces remifentanil and propofol consumption and shortens recovery times in outpatient anaesthesia. Br. J. Anaesth..

[40] Won Y.J., Lim B.G., Kim Y.S., Lee M., Kim H. (2018). Usefulness of surgical pleth index-guided analgesia during general anesthesia: A systematic review and meta-analysis of randomized controlled trials. J. Int. Med. Res..

[41] Bellolio M.F., Gilani W.I., Barrionuevo P., Murad M.H., Erwin P.J., Anderson J.R., Hess E.P. (2016). Incidence of adverse events in adults undergoing procedural sedation in the emergency department: A systematic review and meta-analysis. Acad. Emerg. Med..

[42] Von Delius S., Thies P., Rieder T., Wagenpfeil S., Herberich E., Karagianni A., Frimberger E., Meining A., Ludwig L., Ebert M.P.A. (2009). Auditory evoked potentials compared with bispectral index for monitoring of midazolam and propofol sedation during colonoscopy. Am. J. Gastroenterol..

[43] Bilgi M., Tekelioglu U.Y., Sit M., Demirhan A., Akkaya A., Yildiz I., Kocoglu H. (2015). Comparison of the effects of bispectral index-controlled use of remifentanil on propofol consumption and patient comfort in patients undergoing colonoscopy. Acta Gastro-Enterol. Belg..

[44] Hazewinkel Y., Dekker E. (2011). Colonoscopy: Basic principles and novel techniques. Nat. Rev. Gastroenterol. Hepatol..

[45] Sargin M., Uluer M.S., Şimşek B. (2019). The effect of bispectral index monitoring on cognitive performance following sedation for outpatient colonoscopy: A randomized controlled trial. Sao Paulo Med. J. Rev. Paul. Med..

[46] Krebs E.E., Carey T.S., Weinberger M. (2007). Accuracy of the pain numeric rating scale as a screening test in primary care. J. Gen. Intern. Med..

[47] Aho A.J., Yli-Hankala A., Lyytikäinen L.P., Jäntti V. (2009). Facial muscle activity, Response Entropy, and State Entropy indices during noxious stimuli in propofol—Nitrous oxide or propofol—Nitrous oxide—Remifentanil anaesthesia without neuromuscular block. Br. J. Anaesth..

[48] Apfel C.C., Laara E., Koivuranta M., Greim C.A., Roewer N. (1999). A simplified risk score for predicting postoperative nausea and vomiting: Conclusions from cross-validations between two centers. Anesthesiology.

[49] Darvall J., Handscombe M., Maat B., So K., Suganthirakumar A., Leslie K. (2021). Interpretation of the four risk factors for postoperative nausea and vomiting in the Apfel simplified risk score: An analysis of published studies. Can. J. Anaesth./J. Can. Anesth..

[50] Johnson D.A., Barkun A.N., Cohen L.B., Dominitz J.A., Kaltenbach T., Martel M., Robertson D.J., Boland C.R., Giardello F.M., Lieberman D.A. (2014). Optimizing adequacy of bowel cleansing for colonoscopy: Recommendations from the U.S. multi-society task force on colorectal cancer. Gastrointest. Endosc..

