# Supplementary material for: Adequacy of Anesthesia Guidance for Colonoscopy Procedures
Source: Pharmaceuticals (Basel). 2021 May 14;14(5):464. doi: 10.3390/ph14050464 (PMC8157001; doi:10.3390/ph14050464)
Supplement: Supplementary file 1 [file pharmaceuticals-14-00464-s001.zip › pharmaceuticals-1224603-supplementary.pdf]

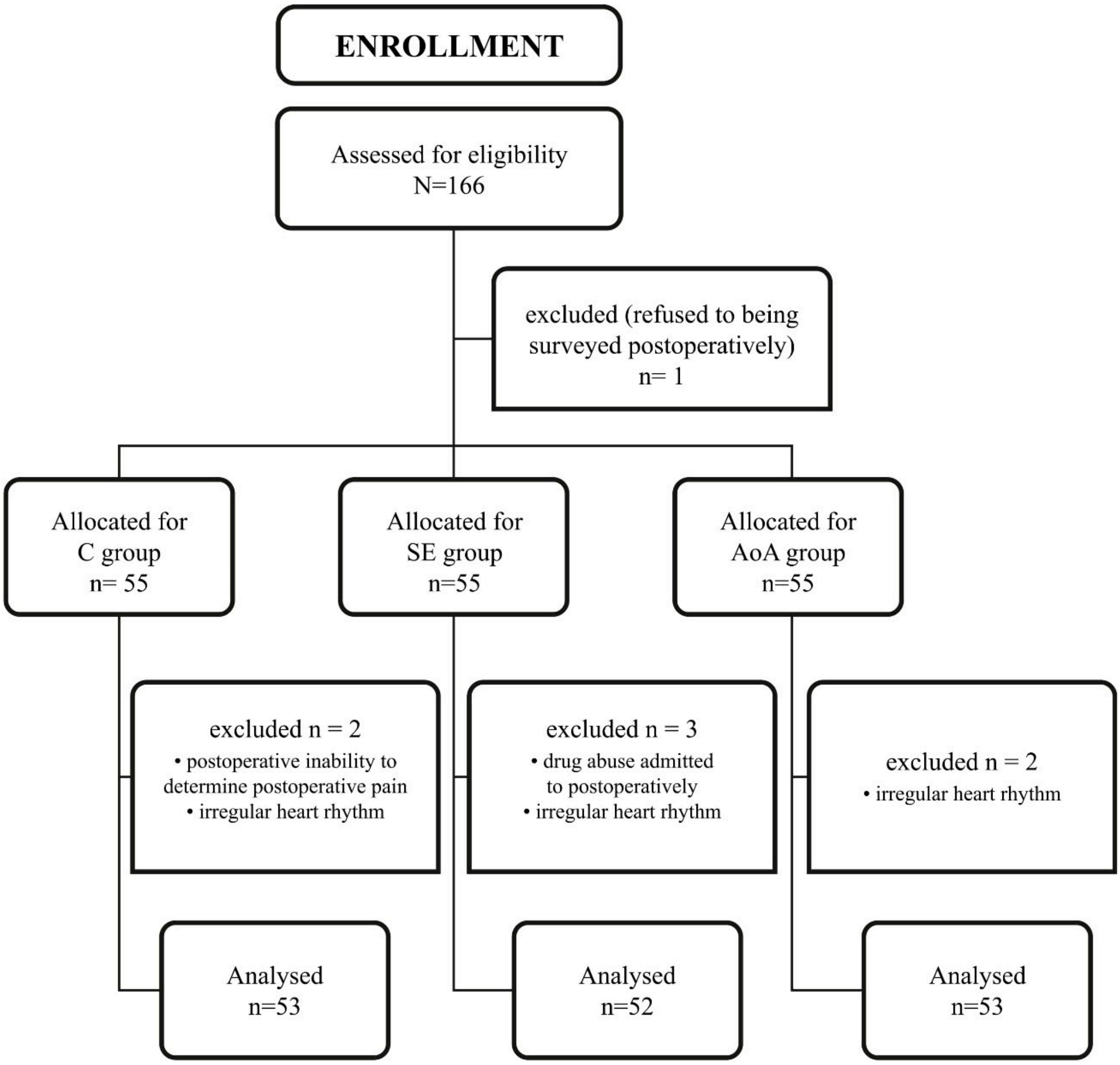

Supplementary Figure 1. Randomization graph—study outline.

Supplementary Table 1. Anthropomertic data of patients in the studied groups: the control group, the SE group, and the AoA group.

**Parameter**	**Total n=158 (100%)**	**Group C n=53 (33.5%)**	**Group SE n=52 (32.9%)**	**Group AoA n=53 (33.5%)**	***p*-value**
AGE (years)	59±14.1<br>62 (18)	60.9±13.9<br>63 (18)	58.7±14.6<br>61.5 (19.5)	57.4±13.9<br>62 (16)	$p = 0.41$
HEIGHT (cm)	167.7±9.4<br>167 (12)	165.9±9.2<br>164 (12)	169.5±8.6<br>170 (12)	167.6±10.1<br>167 (13)	$p = 0.14$
WEIGHT (kg)	76.9±16.5<br>75 (18)	74.5±13.5<br>75 (15)	79.5±18.8<br>75.5 (22.5)	76.9±16.9<br>75 (19)	$p = 0.55$
BMI	27.3±5.3<br>27.3 (5.8)	27.1±5<br>27.4 (4.1)	27.6±6.5<br>27.2 (5.7)	27.2±4.5<br>27.8 (6)	$p = 0.99$
MALE / FEMALE	73 (46.2%) / 85 (53.8%)	23 (43.4%) / 30 (56.6%)	25 (48.1%) / 27 (51.9%)	25 (47.2%) / 28 (52.8%)	$p = 0.87$

Results are presented as a mean ± SD and median (IQR) for quantitative variables and as numbers (percentages) for nominal variables.

p-values were found by a one-way ANOVA test for quantitative variables; p-values were found by an X2 test for nominal variables.

Abbreviations: Group C: control group; Group SE: Entropy SE group; Group AoA: Adequacy of Anaesthesia group; BMI: body mass index; SD: standard deviation; IQR: interquartile range.
